# The gain-of-function GLI1 transcription factor TGLI1 enhances expression of VEGF-C and TEM7 to promote glioblastoma angiogenesis

**DOI:** 10.18632/oncotarget.4248

**Published:** 2015-06-04

**Authors:** Richard L. Carpenter, Ivy Paw, Hu Zhu, Sherona Sirkisoon, Fei Xing, Kounosuke Watabe, Waldemar Debinski, Hui-Wen Lo

**Affiliations:** ^1^ Department of Cancer Biology, Wake Forest University School of Medicine, Winston-Salem, NC 27157, USA; ^2^ Brain Tumor Center of Excellence, Wake Forest University School of Medicine, Winston-Salem, NC 27157, USA; ^3^ Comprehensive Cancer Center, Wake Forest University School of Medicine, Winston-Salem, NC 27157, USA; ^4^ Department of Pharmacology, University of North Carolina School of Medicine, Chapel Hill, NC 27599, USA

**Keywords:** glioblastoma, TGLI1, GLI1, angiogenesis, VEGF-C

## Abstract

We recently discovered that truncated glioma-associated oncogene homolog 1 (TGLI1) is highly expressed in glioblastoma (GBM) and linked to increased GBM vascularity. The mechanisms underlying TGLI1-mediated angiogenesis are unclear. In this study, we compared TGLI1- with GLI1-expressing GBM xenografts for the expression profile of 84 angiogenesis-associated genes. The results showed that expression of six genes were upregulated and five were down-regulated in TGLI1-carrying tumors compared to those with GLI1. Vascular endothelial growth factor-C (VEGF-C) and tumor endothelial marker 7 (TEM7) were selected for further investigations because of their significant correlations with high vascularity in 135 patient GBMs. TGLI1 bound to both VEGF-C and TEM7 gene promoters. Conditioned medium from TGLI1-expressing GBM cells strongly induced tubule formation of brain microvascular endothelial cells, and the induction was prevented by VEGF-C/TEM7 knockdown. Immunohistochemical analysis of 122 gliomas showed that TGLI1 expression was positively correlated with VEGF-C, TEM7 and microvessel density. Analysis of NCBI Gene Expression Omnibus datasets with 161 malignant gliomas showed an inverse relationship between tumoral VEGF-C, TEM7 or microvessel density and patient survival. Together, our findings support an important role that TGLI1 plays in GBM angiogenesis and identify VEGF-C and TEM7 as novel TGLI1 target genes of importance to GBM vascularity.

## INTRODUCTION

Glioblastoma (GBM) is the most frequent and most lethal brain tumor in adults [1]. Prognosis of GBM is poor with median survival of 14 months and less than five percent of patients surviving five years after diagnosis [1, 2]. GBM (grade IV glioma) is more angiogenic, proliferative, and invasive than gliomas at lower grades [3]. Angiogenesis plays a significant role in GBM pathobiology largely because GBM poses a considerable metabolic demand for oxygen delivery and waste removal to sustain its high rates of cell proliferation and metabolism. In line with these notions, GBM vascularity is associated with poor patient prognosis [4, 5]. Consequently, anti-angiogenic therapy for GBM has been developed, most prominently in the form of bevacizumab, a monoclonal antibody targeting vascular endothelial growth factor A (VEGF-A). While bevacizumab treatment initially results in a biological response of tightening the blood-brain barrier, the treatment does not prevent aggressive local and diffusive spread [6–8]. Resistance to anti-VEGF-A therapy suggests there may be other angiogenic factors or pathways that play a role in GBM angiogenesis. For example, VEGF-D was shown to be over-expressed in GBM comparably to VEGF-A [9].

Glioma-associated oncogene homolog 1 (GLI1) is a zinc finger transcription factor serving as the terminal effector of the sonic hedgehog (Shh) signaling pathway [10]. Our lab discovered a novel variant of GLI1, called truncated GLI1 (TGLI1), in which the entire exon III and a portion of exon IV are excluded by alternative splicing [11]. The splicing of this region results in an in-frame deletion of 123 bp (41 amino acids) while retaining all of the functional domains of GLI1 and regulating known GLI1 genes to a similar extent as GLI1 [11]. However, evidence to date indicates that TGLI1 has gained the ability to transcriptionally activate several genes that are not regulated by GLI1 and consequently, gained the propensity to promote tumor migration and invasion. [11–13] Interestingly, TGLI1 was only detectable in cell lines, patient-derived xenografts, and primary specimens of GBM, but undetectable in normal brain or other normal human tissues we had examined [11]. Our most recent study [14] further linked TGLI1 to GBM vascularity as we observed that GBM xenografts with increased TGLI1 being more vascularized; however, the mechanisms underlying this link are still elusive.

Our previous data indicate TGLI1 has increased expression in GBM relative to normal brain tissue and promotes tumor growth with greater vascularity [14]. However, the underlying mechanisms for TGLI1-induced GBM angiogenesis are not fully understood. To help fill this knowledge gap, we subjected TGLI1- and GLI1-expressing tumors to an angiogenesis PCR array and found that TGLI1-expressing tumors showed increased expression of six genes and decreased expression of five genes. Among these identified genes, we subsequently focused on vascular endothelial growth factor-C (VEGF-C) and tumor endothelial marker 7 (TEM7) because of their positive correlations with high vascularity as determined by analyzing NCBI Gene Expression Omnibus (GEO) datasets derived from 135 patient GBMs. We further observed that TGLI1 transcriptionally upregulates VEGF-C and TEM7 gene expression and that TGLI1 levels were significantly associated with microvessel density, VEGF-C levels, and TEM7 levels in a cohort of glioma patients. Both TGLI1 target genes are associated with worse survival of patients with malignant gliomas. In summary, our study provides new insights into the molecular underpinning of GBM abnormal angiogenesis.

## RESULTS

### VEGF-C and TEM7 are upregulated in highly vascularized TGLI1-expressing xenograft tumors

**Figure 1:**
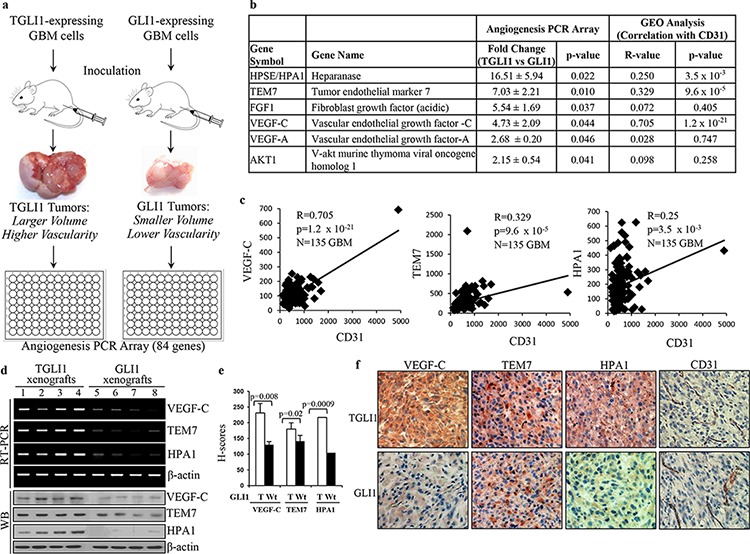
VEGF-C and TEM7 are upregulated in highly vascularized TGLI1-expressing GBM xenografts **a.** TGLI1- and GLI1-expressing GBM xenografts were generated by subcutaneous flank inoculation in nude mice in our previous study [11]. The resulting TGLI1-expressing tumors showed greater size and vascularity than GLI1-expressing tumors. Total RNA was isolated from three TGLI1- and three GLI1-expressing tumors and subjected to RT-PCR and the angiogenesis PCR array. **b.** Six pro-angiogenesis genes were upregulated in TGLI1 tumors compared to GLI1 tumors, as indicated by the angiogenesis PCR array. Analysis of two GEO public datasets (GSE4412 and GSE4271; 135 primary GBMs) showed that HPA1, TEM7 and VEGF-C were positively correlated with the vascular endothelial marker CD31 (*p* < 0.05). **c.** Linear regression plots, derived from the GEO analysis, indicate that VEGF-C, TEM7 and HPA1 significantly correlate with CD31 in patient GBMs (*N* = 135). **d.** RT-PCR and western blotting showed that TGLI1 xenografts expressed higher levels of VEGF-C, TEM7, and HPA1 than those with GLI1. **e.** IHC confirmed that TGLI1 xenografts expressed higher expression of VEGF-C, TEM7, and HPA1 than GLI1 tumors. Histologic scores (H-scores) were computed from percent positivity (A%, A = 1–100) and intensity (B = 0–3) using the equation, H-Score = A × B. Student's *t*-test was used to compute *p*-values. **f.** Representative images of IHC-analyzed xenografts.

In our initial discovery of TGLI1, we observed that TGLI1-expressing GBM xenografts showed greater expression of CD24 with evidence of greater invasiveness compared to GLI1-expressing GBM xenografts [11]. More recently, we showed that TGLI1-expressing GBM xenografts were more proliferative and more vascularized compared to GLI1-expressing tumors [14]. Since angiogenesis is regulated by the balance of pro- and anti-angiogenic factors in a given microenvironment [15], we examined whether TGLI1 regulated expression of angiogenesis-associated genes. To this end, we analyzed xenograft tumors derived from stable TGLI1- or GLI1-expressing GBM cells in our recent studies [11, 14] using an angiogenesis PCR array, which detects expression of 84 human genes related to angiogenesis (Fig. 1a). The results indicated that six genes were expressed at significantly higher levels, while five genes expressed at lower levels, in TGLI1-expressing tumors than GLI1-expressing tumors (Fig. 1b and Tables S1 and S2 in Supplementary Information). To provide an additional filter to select genes for further investigations, we analyzed NCBI GEO public datasets (GSE4271 [16] and GSE4412 [17]; 135 primary GBMs) to determine the degrees to which these genes are correlated with CD31, a vascular endothelial marker, in patient GBMs. As indicated in Fig. 1b, expression of HPA1, TEM7 and VEGF-C, but not FGF1, VEGF-A or AKT1, were positively correlated with levels of CD31 (*p* < 0.05). Linear regression plots in Fig. 1c indicate that TEM7, VEGF-C and HPA1 expression significantly and positively correlate with CD31 in GBM samples.

Next, we confirmed the positive associations of TGLI1 with TEM7 and VEGF-C at the mRNA and protein levels using GBM xenografts (Fig. 1e). These associations were further validated using immunohistochemistry (IHC) as the TGLI1 xenografts showed higher expression of VEGF-C, TEM7, and HPA1 (Fig. 1e). Representative images of IHC-analyzed xenografts are shown in Fig. 1f. Taken together, the results in Fig. 1 indicate that TGLI1 may promote GBM neo-vascularity by upregulating the expression of several angiogenic factors.

### TGLI1 upregulates expression of VEGF-C and TEM7

**Figure 2:**
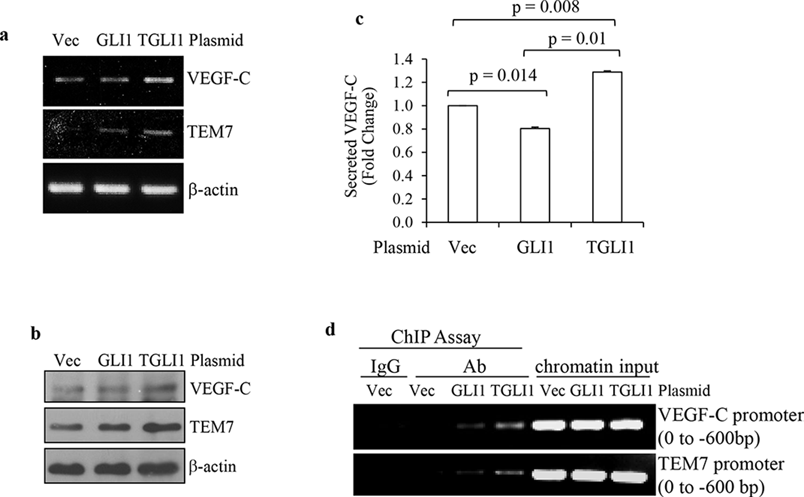
TGLI1 upregulates VEGF-C and TEM7 gene expression **a** and **b.** Transient expression of TGLI1 enhances expression of VEGF-C and TEM7 in GBM cells. TGLI1, GLI1, or an empty vector were transfected into U87MG GBM cells and the total RNA or total protein was isolated from these cells and subjected to RT-PCR (a) or western blotting (b), respectively. **c.** ELISA shows that the TGLI1-overexpressing GBM cells secreted more VEGF-C into the conditioned medium compared to the cells with GLI1 or control vector. **d.** The ChIP assay shows that TGLI1 bound to the VEGF-C and TEM7 gene promoters more strongly than GLI1. IgG was used as negative control for an antibody recognizing both TGLI1 and GLI1. Chromatin input was used in PCR as loading controls.

We next aimed to determine whether TGLI1 can directly upregulate VEGF-C and TEM7. Of note, the direct link between TGLI1 and HPA1was reported in our recent study [14]; therefore, we subsequently focused our efforts on VEGF-C and TEM7 in this study. VEGF-C is a member of the VEGF family that is regarded as a trophic factor for neural progenitors in the vertebrate embryonic brain [18]. VEGF-C can bind to and activate VEGFR3, leading to lymphangiogenesis [19, 20]. VEGF-C can also bind to and activate VEGFR2, leading to angiogenesis and cell growth. [19–21] TEM7 is a type I transmembrane protein with a large extracellular domain [22] whose biological function is still unclear. TEM7 has been shown to interact with extracellular matrix components and may play a role in cell migration, invasion, and capillary morphogenesis [23–26]. We transiently transfected U87MG cells with either TGLI1 or GLI1, or the control empty vector. Total RNA was subjected to RT-PCR and we observed greater mRNA levels of VEGF-C and TEM7 in TGLI1-expressing cells compared to GLI1- or vector-expressing cells (Fig. 2a). Fig. 2b shows that the protein levels of VEGF-C and TEM7 were also greater in TGLI1-expressing cells compared to GLI1- or vector-expressing cells. The conditioned medium of these cells was collected and subjected to VEGF-C ELISA to determine if TGLI1 made cells secrete more VEGF-C than GLI1. The results showed that TGLI1-expressing cells had significantly higher secretion of VEGF-C than GLI1- or vector-expressing cells (Fig. 2c). We then conducted the chromatin immunoprecipitation (ChIP) assay and found that TGLI1 bound to both VEGF-C and TEM7 gene promoters more strongly than GLI1 (Fig. 2d). The enrichment of TGLI1 occurred within the first 600 bp of the gene promoters, which was identified using different primer sets that scanned the proximal 3 kb of these promoters. Together, these results indicate the novel finding that TGLI1 can upregulate VEGF-C and TEM7 expression via direct binding to the respective gene promoters.

### VEGF-C contributes to TGLI-induced angiogenesis

**Figure 3:**
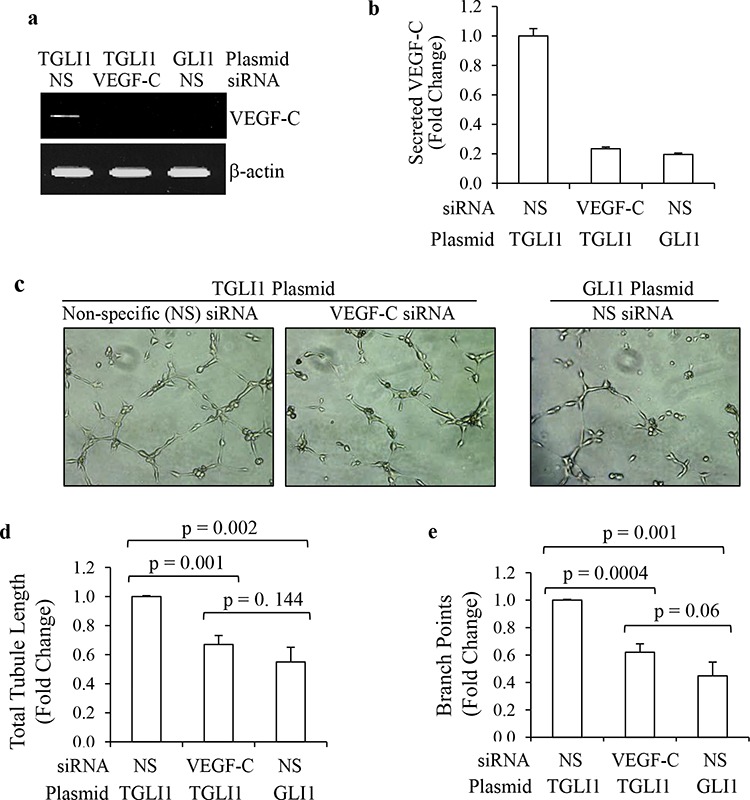
TGLI1 promotes *in vitro* angiogenesis via VEGF-C **a, b.** VEGF-C knockdown was effective. U87MG cells expressing TGLI1 or GLI1 were transfected with siRNA directed to VEGF-C or non-specific (NS) control siRNA. Total RNA was isolated and subjected to RT-PCR (a). Conditioned medium was subjected to VEGF-C ELISA assay (Bb). Student *t*-test was used to calculate *p*-values. **c–e.** Tubule formation assay showed that VEGF-C expression knockdown of VEGF-C reduced the ability of TGLI1-expressing GBM cells to promote angiogenesis of human brain microvascular endothelial cells. Representative images are shown in (c). Total tubule length was determined and shown in (d). Number of branch points was counted and shown in (e). Student *t*-test was used to calculate *p*-values.

Next, we studied whether VEGF-C is a significant contributor to TGLI1-induced angiogenesis. As shown in Fig. 3a, knockdown using siRNA directed to VEGF-C effectively reduced VEGF-C levels in TGLI1-expressing GBM cells. Non-specific (NS) siRNA served as a negative control. Fig. 3b shows that VEGF-C knockdown significantly reduced TGLI1-induced secretion of VEGF-C. Next, we determined the effect of VEGF-C knockdown in GBM cells on *in vitro* angiogenesis of microvascular endothelial cells. To this end, TGLI1- or GLI1-expressing U87MG cells were transfected with either control or VEGF-C siRNA. Conditioned medium from these cells was placed on human brain microvascular endothelial cells, which were monitored for tubule formation (Fig. 3c). Knockdown of VEGF-C in TGLI1-expressing cells significantly reduced both total tubule length and branch points of human brain microvascular endothelial cells compared to control siRNA (Figs. 3c–3e). These data suggest that VEGF-C may play an important role in TGLI1-induced angiogenesis.

### TEM7 contributes to TGLI1-induced angiogenesis

**Figure 4:**
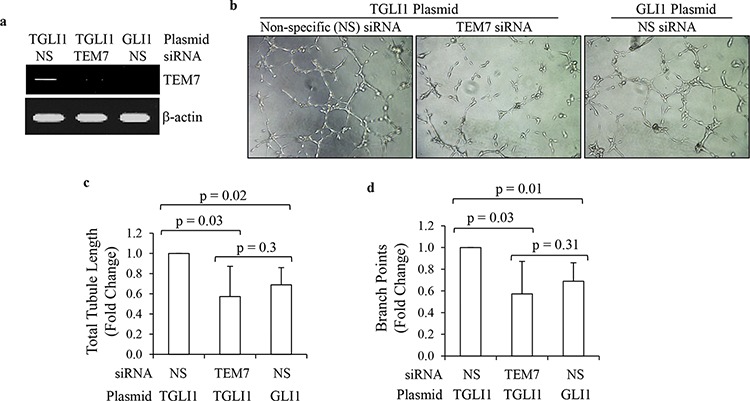
TGLI1 promotes *in vitro* angiogenesis via TEM7 **a.** TEM7 knockdown was efficient. U87MG cells expressing TGLI1 or GLI1 were transfected with TEM7 or control NS siRNA. Total RNA was isolated and subjected to RT-PCR. **b–d.** Tubule formation assay showed that TEM7 expression knockdown reduced the propensity of TGLI1-expressing GBM cells to promote angiogenesis of human brain microvascular endothelial cells. Representative images are shown in (b). Total tubule length was calculated and shown in (c). Number of branch points was counted and shown in (d). Student *t*-test was used to calculate *p*-values.

We next determined whether TEM7 is a significant contributor to TGLI1-induced angiogenesis. Knockdown of TEM7 using siRNA significantly reduced TEM7 expression in TGLI1-expressing GBM cells (Fig. 4a). To determine the contribution of TEM7 to TGLI1-induced *in vitro* angiogenesis, we collected conditioned medium from TGLI1-expressing GBM cells with or without TEM7 knockdown and incubated the conditioned media on human brain microvascular endothelial cells and monitored the extent of tubule formation (Fig. 4b). Conditioned medium from TGLI1-expressing cells with knockdown of TEM7 induced significantly lower total tubule lengths and branch points compared to TGLI1-expressing cells with non-specific control siRNA (Figs. 4c–4e). These results suggest TEM7 is also a potentially important mediator of TGLI1-induced angiogenesis.

### TGLI1 levels correlate with microvessel density in patients with gliomas

**Figure 5:**
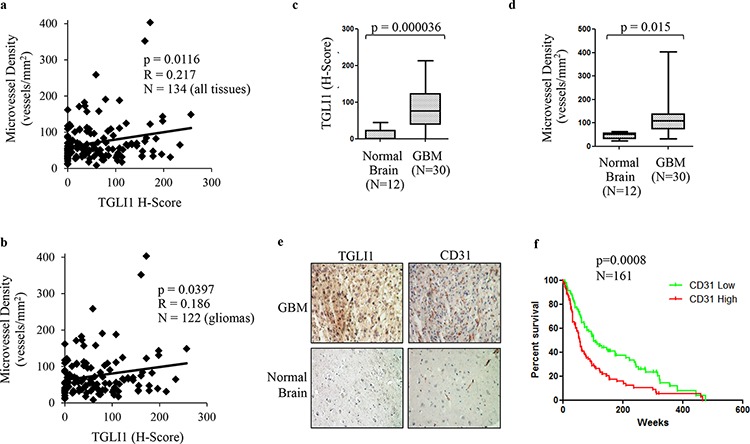
TGLI1 levels are positively associated with microvessel density in patient gliomas **a.** TGLI1 expression positively correlates with microvessel density. A tumor microarray with 134 tissues (122 gliomas across all grades and 12 normal brain tissues) was subjected to IHC with antibodies for TGLI1 (for H-score) and CD31 (for microvessel density). Pearson Correlation was used. **b.** TGLI1 positively correlated with microvessel density in 122 glioma tissues across all grades. Pearson Correlation was used. **c.** TGLI1 was expressed at higher levels in GBM tumors compared to normal brain tissues. Student *t*-test was used. **d.** Microvessel density was higher in GBM tumors than in normal brain tissues. Student *t*-test was used. **e.** Representative images of IHC-analyzed tissues. **f.** GEO data mining analysis of 161 malignant gliomas indicated that high CD31 expression predicted poor patient survival. Red: tumors with high CD31. Green: tumors with low CD31.

We previously published the development of rabbit polyclonal TGLI1-specific antibodies that do not cross-react with GLI1 and we have used the antibodies to selectively detect TGLI1 via immunohistochemistry (IHC) [14]. Using the TGLI1-specific and CD31 antibodies in IHC, we assessed whether TGLI1 expression is related to microvessel density in a cohort of glioma patients consisting of 134 tissues (122 glioma across all grades and 12 normal brain). CD31 signals mark vascular endothelial cells and were used to derive microvessel density. As shown in Fig. 5a, we observed a statistically significant correlation between TGLI1 expression and microvessel density in this cohort. We next limited our analysis to gliomas only (grades I-IV) and the statistically significant correlation between TGLI1 levels and microvessel density remained (Fig. 5b). We also observed that TGLI1 levels were significantly higher in GBM tumors compared to normal brain (Fig. 5c). Additionally, microvessel density was significantly higher in GBM samples compared to normal brain (Fig. 5d), consistent with the pro-angiogenic nature of GBM. Fig. 5e shows a representative case of GBM with high nuclear TGLI1 expression and high vascularity (visualized with CD31 IHC) as well as a normal brain tissue with low levels of TGLI1 and vascularity. Furthermore, GEO analysis of 161 malignant gliomas indicated that high CD31 expression (indicative of high vascularity) predicted poor patient survival (*p* = 0.0008; Fig. 5f). Cumulatively, these data further support an important role that TGLI1 plays in GBM angiogenesis.

### TGLI1 levels correlate with VEGF-C in patients with GBM

**Figure 6:**
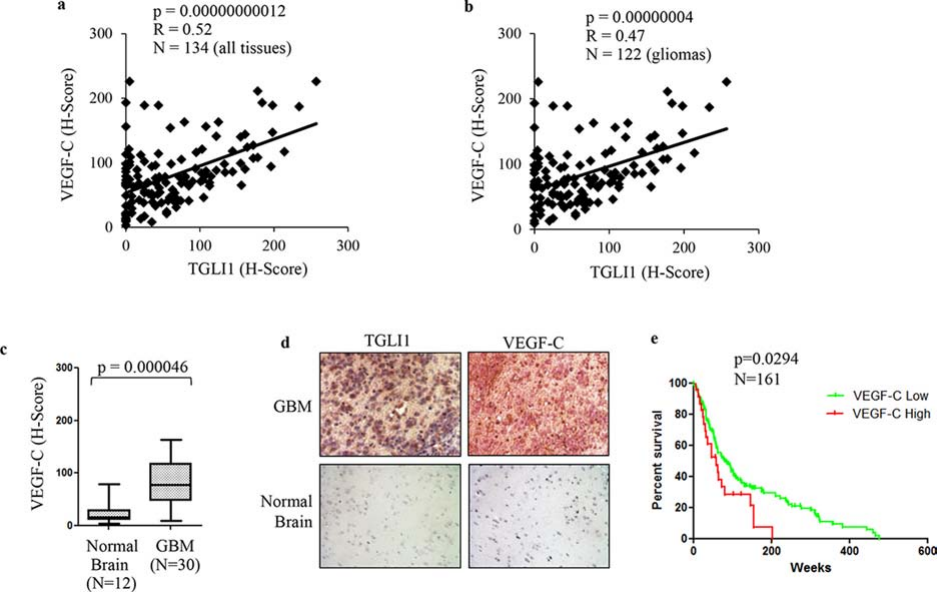
High TGLI1 levels are associated with increased VEGF-C expression in patient gliomas **a.** TGLI1 expression positively correlates with VEGF-C expression. The tumor microarray used in Fig. 5 was additionally stained for VEGF-C. Pearson Correlation was used to correlate TGLI1 with VEGF-C. **b.** TGLI1 positively correlated with VEGF-C in 122 glioma tissues across all grades. Pearson Correlation was used. **c.** VEGF-C was expressed at higher levels in GBMs compared to normal brain tissues. Student *t*-test was used. **d.** Representative images of IHC-analyzed tissues. **e.** GEO data mining of 161 malignant gliomas showed that high VEGF-C expression predicted poor patient survival. The log-rank survival analysis was used. Red: tumors with high VEGF-C. Green: tumors with low VEGF-C.

Considering that we found VEGF-C as a novel direct target of TGLI1, we next wanted to assess the relationship of VEGF-C and TGLI1 in patients. Therefore, we assessed the expression of both VEGF-C and TGLI1 in 122 patient gliomas across all grades (I-IV) plus 12 normal brain tissues. The tissues were analyzed by IHC with antibodies for VEGF-C or TGLI1 followed by histological scoring. We observed a strongly significant correlation between TGLI1 levels and VEGF-C levels in these tissues (Fig. 6a). We next limited our analysis to gliomas only (I-IV) and the statistically significant correlation between TGLI1 and VEGF-C persisted (Fig. 6b) indicating that VEGF-C expression is associated with TGLI1 levels in tumors. Consistent with TGLI1, we also found that VEGF-C levels were significantly higher in GBM tumors compared to normal brain tissue (Fig. 6c). Fig. 6d shows TGLI1 and VEGF-C expression for a patient with high TGLI1 and high VEGF-C expression whereas we did not detect any positive signal in normal brain tissue. Furthermore, GEO analysis of 161 malignant gliomas indicated, for the first time, that high VEGF-C expression predicted shortened patient survival (*p* = 0.0294; Fig. 6e). These data indicate TGLI1 levels correlate with VEGF-C in patient GBMs and suggest that TGLI1 may contribute to poor clinical outcome of GBM patients through upregulating VEGF-C expression.

### TGLI1 levels correlate with TEM7 in patient GBMs

**Figure 7:**
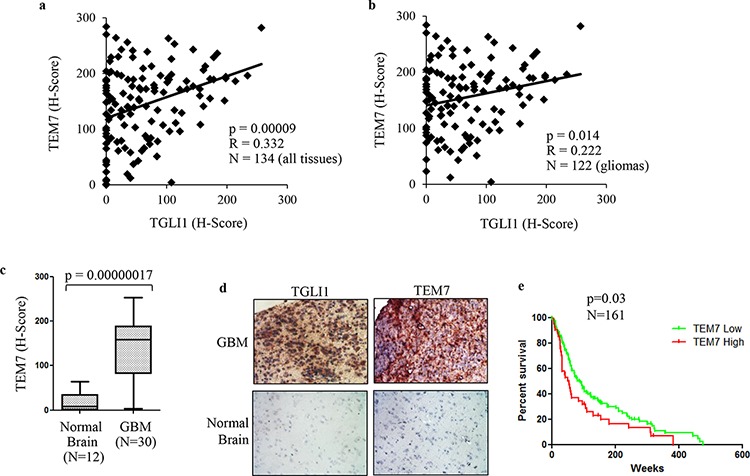
TGLI1 levels are positively associated with TEM7 levels in a cohort of patient gliomas **a.** TGLI1 expression positively correlates with VEGF-C expression. The tumor microarray used in Fig. 5 was additionally stained for VEGF-C. Pearson Correlation was used to correlate TGLI1 with VEGF-C. **b.** A positive correlation was found between TGLI1 and TEM7 in 122 glioma tissues across all grades. Pearson Correlation was used. **c.** TEM7 levels were higher in GBM tumors than in normal brain tissues. Student *t*-test was used. **d.** Representative images of IHC-analyzed tissues. **e.** GEO data mining of 161 malignant gliomas indicated that tumors with high TEM7 expression were associated with worse patient survival than those with low TEM7 expression. The log-rank survival analysis was used. Red: tumors with high TEM7. Green: tumors with low TEM7.

To further investigate the relationship between TGLI1 and TEM7, we determined their expression levels in 122 patient gliomas with varying grades (I-IV) plus 12 normal brain tissues using IHC. The tissues underwent IHC with antibodies for TEM7 or TGLI1 following by histological scoring. Levels of TEM7 were significantly correlated with levels of TGLI1 (Fig. 7a). When we limited our analysis to gliomas only, the significant correlation between TGLI1 and VEGF-C was still observed (Fig. 7b) indicating TEM7 levels are associated with TGLI1 in tumors. Accordingly, we also observed significantly higher levels of TEM7 in GBM tumors compared to normal brain (Fig. 7c). As shown in Fig. 7d, a representative GBM expressed high levels of TGLI1 and TEM7 whereas a normal brain tissue lacked expression of both proteins. GEO analysis further revealed the novel finding that high TEM7 expression predicted poor survival of 161 patients with malignant gliomas (*p* = 0.03; Fig. 7e). Results in Fig. 7 indicate TGLI1 expression is positively linked to TEM7 expression in patient GBMs and also suggest that TGLI1 potentially contributes to poor clinical outcome of GBM patients through enhancing TEM7 expression, similarly to VEGF-C.

## DISCUSSION

GBM is primarily characterized by infiltration, high rates of proliferation, and abnormal neo-vascularization. Efforts have been invested into understanding molecular underpinning of these hallmarks of GBM. However, the biological understanding of GBM tumors is still insufficient, which contributes to the lack of effective treatments for the disease. In this study, we report the following novel findings: (1) TGLI1, an alternatively spliced variant of GLI1 discovered in our laboratory, is highly expressed in GBM specimens but undetectable in normal brain; (2) TGLI1 promotes GBM angiogenesis; (3) TGLI1 transcriptionally activates expression of two angiogenic factors, VEGF-C and TEM7, leading to tumor angiogenesis; (4) the associations of TGLI1 with VEGF-C and TEM7 are observed in cell lines, xenografts and specimens of GBM; (5) expression of TGLI1, VEGF-C and TEM7 is associated with increased vascularity of GBM specimens; and (6) GBM patients with higher levels of VEGF-C, TEM7 or CD31 (a vascular endothelial marker) in their tumors had worse survival than those with low levels. With the aforementioned evidence, this study sheds new light into the molecular underpinnings of high vascularity characteristic of GBM.

VEGF-C has been functionally linked to both angiogenesis and lymphangiogenesis [19, 27–29]. VEGF-C and its receptors (VEGFR2 and VEGFR3) have been detected in GBM [30–32]. However, how VEGF-C is upregulated in GBM has remained poorly understood. Whether VEGF-C plays any role in GBM vascularity is elusive. In addressing these knowledge gaps, the current study provides evidence suggesting an important role that TGLI1 may play in upregulating VEGF-C expression in GBM and a vital role for VEGF-C as an angiogenic factor in GBM. Furthermore, whether VEGF-C is an independent predictor for GBM patient survival is currently unknown. Importantly, our analysis of GEO datasets showed that high VEGF-C expression predicted poor survival of 161 malignant gliomas. This observation is in line with a previous report showing that co-expression of VEGF-C with MMP-1 in gliomas was associated with decreased survival [33]. Additional analysis of a larger glioma patient cohort is warranted to solidify the role of VEGF-C as an independent indicator for poor survival of glioma patients.

Our data indicate VEGF-C has higher expression in GBM compared to normal brain tissue and VEGF-C is associated with reduced GBM patient survival. We have previously shown that TGLI1 also directly upregulates VEGF-A [12]. VEGF-A-targeted therapy (primarily Avastin) has shown relatively disappointing results clinically. Our findings that TGLI1 is also expressed in GBM, but not normal brain [11, 14], and drives expression of both VEGF-A and VEGF-C may indicate VEGF-C is a possible mechanism for resistance to VEGF-A-targeted therapy. In the future, it would be beneficial to study Avastin-resistant tumors to assess the levels of other VEGF isoforms, notably VEGF-C and VEGF-D, which has also been shown to be expressed in GBM [34].

Since the central nervous system does not have a traditional lymphatic system, VEGF-C function in gliomas is likely to promote angiogenesis as well as survival and growth of cancer cells expressing VEGF receptors. Interestingly, glioma stem cells (GSCs) have been shown to be located in the perivascular niche [35, 36]. VEGF-C has been shown to be a trophic factor for neural progenitor cells [18], possibly indicating that VEGF-C may also play a role in GSCs. GSCs promote tumorigenesis, promote resistance to GBM therapies, and are associated with poor patient survival [37–42]. While activation of the hedgehog-GLI1 pathway is associated with GSCs [43], it is currently unknown whether TGLI1 is expressed in or promotes the formation of GSCs. In light of these notions, future studies are needed to explore whether TGLI1 plays a role in GSCs and whether this occurs through the ability of TGLI1 to transcriptionally activate stemness genes.

TEM7 can be detected in the endothelium of multiple types of cancer [25, 44–46], some neuronal populations of the vertebrate brains [26, 47], and also in tumors [23]. TEM7 is a type I transmembrane protein with a large extracellular domain, but has been shown to express intracellularly and be secreted [22]. Although its biological function is still unclear, TEM7 has been shown to interact with extracellular matrix components and may play a role in cell migration, invasion, and capillary morphogenesis [23–26]. TEM7 has been linked to neuronal stem cells [26, 47]. High expression of TEM7 in osteogenic sarcomas was associated with poor survival and high-grade tumors [23]. However, the extent of TEM7 expression in GBM is not known and the relationship between TEM7 and GBM patient survival is presently unknown. In this study, we report that TEM7 is expressed in gliomas whose expression is upregulated by TGLI1, and that TEM7 is potentially an important mediator of GBM angiogenesis and also a predictor for poor survival of glioma patients. These important observations warrant future investigations into the precise biological functions of tumoral TEM7 in the context of GBM angiogenesis and potentially other malignant phenotypes of GBM. For example, TEM7 may potentially play a role in GBM invasiveness and GSCs. The invasion front of GBMs is also thought to be another niche wherein GSCs are located and expand the tumor by invasion of the surrounding tissue [48]. Since TEM7 has been shown to play a role in cell invasion [24, 25, 49] and neuronal stem cells [26, 47], it is an important task to investigate whether TEM7 promotes GBM invasiveness and potentially the GSC niche in this compartment.

Since our discovery of TGLI1 in 2009, we have uncovered several important properties and functions of TGLI1. We showed that TGLI1 is highly expressed in cell lines and specimens of GBM and breast cancer, but undetectable in normal counterparts [11–14]. We further reported that TGLI1 behaves as a gain-of-function GLI1 transcription factor that regulates not only known GLI1 target genes but also additional genes not targeted by GLI1, including CD24, VEGF-A, VEGFR2, and HPA [11–14]. Consequently, TGLI1 has been demonstrated to promote tumor cell migration, invasion, proliferation, and angiogenesis [11–14]. The mechanism by which TGLI1 can regulate genes that regulated by GLI1 is still unknown. To address this knowledge gap, we are currently undertaking studies to determine whether: 1) TGLI1 recognizes DNA motifs that are not bound to GLI1, leading to transcriptional upregulation of VEGF-C and TEM7; and 2) TGLI1 interacts with transcription co-factors that GLI1 does not interact with and thereby upregulates VEGF-C and TEM7. The mechanism by which TGLI1 mediates GBM angiogenesis has remained unclear, we undertook the current study using an unbiased approach and found that TGLI1 enhances expression of VEGF-C and TEM7, leading to abnormal neo-angiogenesis often observed in GBM specimens. It is worth noting that the angiogenesis gene profiling study identified five genes to be down-regulated with TGLI1 expression. In line with this interesting observation, our earlier DNA microarray results indicated that 23 genes were down-regulated by TGLI1. In light of these interesting observations, a future task is to explore the role of TGLI1 as a transcription repressor. Since microRNAs (miRNAs) can repress gene expression, it is also a plausible future direction to explore whether TGLI1 represses gene transcription by upregulating expression of some miRNAs.

In summary, the findings reported in this study and our previous studies together have laid the foundation for future efforts to investigate: 1) the role of TGLI1 in cancer stem cells, 2) the specific splicing mechanisms that synthesize TGLI1 in tumor cells but not in normal counterparts, 3) the role of TGLI1 in gene repression and miRNA expression, 4) the value of TGLI1 as a prognostic indicator in human cancers, 5) the potential to pharmacologically target TGLI1, and 6) the involvement of VEGF-C and TEM7 in GBM angiogenesis and other malignant phenotypes.

## MATERIALS AND METHODS

### Reagents and cell culture

All chemicals were purchased from Sigma (St. Louis, MO) unless otherwise stated. Expression vectors, pCMV-Tag2b, pCMV-Tag2b-GLI1, and pCMV-Tag2b-TGLI1, were generated in our laboratory [11]. U87MG cells were purchased from American Type Culture Collection (ATCC) and were maintained in DMEM (Gibco) supplemented with 10% FBS (Gemini) and 1% penicillin/streptomycin (Gibco). The siRNAs for TEM7 (GUGCCAGAAUCUCGGCGAA) and VEGF-C (CCAAUUACAUGUGGAAUAA) were purchased from Thermo Scientific (Lafayette, CO). The non-targeting control siRNA was obtained from Bioneer (Alameda, CA) with the sequence of CCUACGCCACCAAUUUCGU(dTdT).

### Angiogenesis PCR array

Total RNA was isolated from the xenograft tumors (three per group) using the SV Total RNA Isolation System (Promega). Complementary DNA (cDNA) was produced from 1 μg total RNA using the RT^2^ first strand kit (SABioscience). The cDNA was then analyzed on a human angiogenesis RT^2^ Profiler PCR Array (SABioscience) using a Stratagene Mx3005p qPCR system (Stratagene). The PCR array detected 84 genes related to angiogenesis and is specific to human genes without cross-reactivity with genes of other species. The genes found to be significantly different (> 2-fold; *p* < 0.05) between U87MG-TGLI1 and U87MG-GLI1 xenografts were further analyzed using RT-PCR.

### GEO analysis of gene expression profiles and patient overall survival

We compiled a DNA microarray dataset of 185 patient malignant gliomas, with 135 being GBM, from GEO (GSE4271 [16] and GSE4412 [17]). The datasets were normalized using MAS5.0 and each microarray was centered to the median for all probes, as we previously conducted [50]. We performed Pearson Correlation to determine the relationships between selected genes using Microsoft Excel. Of the 185 total patients included in this compiled cohort, 161 patients had survival data accessible. Using data from these 161 patients (114 GBMs and 47 grade III gliomas), we performed the log-rank survival analysis for CD31, VEGF-C, and PLXDC1 (TEM7) genes using Prism GraphPad version 5. A histogram for the expression of each gene was used to determine a rational cut point for high or low expression of each gene for survival analysis. The cutoffs used for CD31, VEGF-C and TEM7 were 50%, 86% and 75% respectively.

### RT-PCR

Total RNA isolation was conducted using SV Total RNA Isolation system (Promega) and RT was done with Superscript III First-Strand cDNA synthesis system (Invitrogen). The following primers were used for PCR: TEM7: Forward 5′-GGAGTGGATGGACTATGGCT-3′ Reverse 5′-AGGGAGGAGGAGGTAGTGGT-3′ VEGF-C: Forward 5′-GGCTGGCAACATAACAGAGA-3′ Reverse 5′-GTGGCATGCATTGAGTCTTT-3′ β-actin: Forward 5′-GGCGGCACCACCATGTACCC-3′ Reverse 5′-AGGGGCCGGACTCGTCATACT-3′.

### Immunoblotting

Immunoblotting was done as we described previously. [14] Antibodies used include VEGF-C (R&D), TEM7 (Novus Biologicals), HPA1 (Santa Cruz; H-80) and β-actin (Sigma).

### Immunohistochemistry and tissue microarray

Tumor sections were deparaffinized and immunohistochemistry (IHC) was conducted as we described previously [14]. Tissue microarrays (US Biomax; GL2083) were deparaffinized followed by probing with antibodies for goat polyclonal VEGF-C antibody (R&D; AF752; 1:50), mouse monoclonal TEM7 antibody (Novus Biologicals; NB 100–56557; 1:75), rabbit polyclonal CD31 antibody (Thermo Scientific, Clone JC/70A, ready-to-use), and rabbit polyclonal HPA1 antibody (Santa Cruz; H-80; 1:25). TGLI1 antibodies were developed by us as we previously described [14]. Histologic scores (H-scores) were computed from percent positivity (A%, A = 1–100) and intensity (B = 0–3) using the equation, H-Score = A × B. MVD was calculated by determining the area (mm^2^) of each tissue core on the tissue microarray following by manual counting of vessels within the entire tissue core. MVD was then calculated as the vessels/mm^2^.

### ELISA

Cells were seeded in 24-well plates and incubated in EBM-2 basal medium at 37°C for 24 hrs. Conditioned medium was then collected and centrifuged at 1200 g for 10 min. The supernatants were then subjected to Enzyme-linked immunosorbent assay (ELISA) using a VEGF-C ELISA kit (R&D) performed according to manufacturer's instructions. Absorbance was measured using the Synergy HT Multi-mode microplate reader (BioTek) at 540 nm with 450 nm measurements serving as the background normalization. Concentrations were computed with reference to a standard curve per manufacturer's instructions for the ELISA kit.

### Chromatin Immunoprecipitation (ChIP)

This was performed using a ChIP Assay kit (Upstate, Billerica, MA) as we described previously [14]. A GLI1 antibody (Santa Cruz, H-300) that recognizes the COOH-terminal region present in both GLI1 and TGLI1 proteins was used for immunoprecipitation of cells expressing TGLI1, GLI1, or vector. The following primers were used to detect presence of the VEGF-C gene proximal promoter (within −600 bp): 5′-GGAGGACAAGAACTCGGGA-3′ and 5′-TGCCTGCGCTTATGTGAGAGA-3′. The following primers were used to amplify TEM7 gene proximal promoter (within −600 bp): 5′-GTGGAGGGATAAGGTG GAGT-3′ and 5′-GAGAACCCCTAGAAGCATCA-3′.

### Tubule formation assay

This was performed using the *In Vitro* Angiogenesis kit (Trevigen), as we previously described [14]. Briefly, 5000 human brain microvascular endothelial cells were seeded into each coated well. Conditioned medium was collected after incubation with tumor cells for 24 hours and then added to the endothelial cells for 4–6 hrs. Endothelial cells were then photographed under a light microscope. Images were analyzed for tubule formation, which was quantified by measuring total tubule length and total number of branch points using ImageJ software. Experiments were completed in triplicate.

### Statistical analysis

Data are presented as mean ± SE. The student *t*-test and Pearson Correlation were performed as necessary using STATISTICA (StatSoft) and Microsoft Excel. For survival correlations, the log-rank survival analysis was performed using Prism GraphPad version 5. Significance was set at *p* < 0.05.

## SUPPLEMENTARY TABLES


