# Supplementary material for: The gain-of-function GLI1 transcription factor TGLI1 enhances expression of VEGF-C and TEM7 to promote glioblastoma angiogenesis
Source: Oncotarget. 2015 Jun 4;6(26):22653–65. doi: 10.18632/oncotarget.4248 (PMC4673189; doi:10.18632/oncotarget.4248)
Supplement: Supplementary file 1 [file oncotarget-06-22653-s001.pdf]

SUPPLEMENTARY TABLES

Supplementary Table S1. Angiogenesis-associated genes down-regulated by TGLI1 compared to GLI1 in GBM xenografts. TGLI1- and GLI1-expressing GBM xenografts were subjected to a PCR angiogenesis array as described in the Materials and Methods. This table shows five genes that were significantly down-regulated by TGLI1 compared to GLI1. The Student's ***t***-test was conducted to compute ***p***-values

Gene Symbol	Gene Name	Fold Change(TGLI1 vs GLI1)	*p*-value
ANGPT1	Angiopoietin 1	0.41 ± 0.04	0.018
ANGPTL3	Angiopoietin-like 3	0.72 ± 0.04	0.035
CCL2	Chemokine (C-C motif) ligand 2	0.49 ± 0.04	0.004
EREG	Epiregulin	0.25 ± 0.09	0.033
IGF1	Insulin-like growth factor 1	0.09 ± 0.07	0.025

Supplementary Table S2. Angiogenesis-associated Genes unaltered by TGLI1 compared to GLI1. TGLI1- and GLI1-expressing GBM xenografts were subjected to a PCR angiogenesis array as described in the Materials and Methods. The Student's ***t***-test was conducted to compute ***p***-values. This table shows genes that had no significant differences in expression levels between the TGLI1 and GLI1 xenografts

Gene Symbol	Gene Name	Fold Change (TGLI1 vs. GLI1)	*p*-value
ANG2	Angiopoietin 2	0.97 ± 0.30	0.94
ANGPTL4	Angiopoietin-like 4	10.28 ± 6.00	0.06
ANPEP	Alanyl (membrane) aminopeptidase	8.05 ± 4.38	0.05
BAI1	Brain-specific angiogenesis inhibitor 1	0.37 ± 0.18	0.25
CCL11	Chemokine (C-C motif) ligand 11	8.28 ± 1.70	0.08
CDH5	Cadherin 5, type 2 (vascular endothelium)	1.64 ± 0.66	0.68
COL18A1	Collagen, type XVIII, alpha 1	0.63 ± 0.12	0.16
COL4A3	Collagen, type IV, alpha 3 (goodpasture antigen)	9.23 ± 4.56	0.10
CXCL1	Chemokine (C-X-C motif) ligand 1	0.65 ± 0.22	0.36
CXCL3	Chemokine (C-X-C motif) ligand 3	1.93 ± 0.69	0.22
CXCL5	Chemokine (C-X-C motif) ligand 5	6.59 ± 2.88	0.10
TYMP	Thymidine phosphorylase	3.02 ± 0.95	0.08
S1PR1	Sphingosine-1-phosphate receptor 1	0.89 ± 0.02	0.30
EFNA1	Ephrin-A1	2.83 ± 1.45	0.43
EFNA3	Ephrin-A3	0.67 ± 0.22	0.49
EFNB2	Ephrin-B2	1.41 ± 0.23	0.12
EGF	Epidermal growth factor	1.24 ± 0.16	0.47
EPHB4	EPH receptor B4	1.31 ± 0.41	0.75
FGF2	Fibroblast growth factor 2 (basic)	0.84 ± 0.11	0.46
FIGF	C-fos induced growth factor (vascular endothelial growth factor D)	4.94 ± 3.87	0.41
HAND2	Heart and neural crest derivatives expressed 2	1.10 ± 0.24	0.93
HGF	Hepatocyte growth factor	2.40 ± 1.33	0.42
HIF1α	Hypoxia inducible factor 1, alpha subunit	1.30 ± 0.30	0.44
ID1	Inhibitor of DNA binding 1	0.66 ± 0.17	0.15
ID3	Inhibitor of DNA binding 13	1.63 ± 0.60	0.43
IL-1B	Interleukin-1 beta	1.58 ± 0.22	0.20
IL-6	Interleukin-6	1.06 ± 0.26	0.99
IL-8	Interleukin-8	3.92 ± 1.42	0.09
ITGAV	Integrin, alpha V	1.26 ± 0.32	0.56
ITGB3	Integrin, beta 3	1.10 ± 0.45	0.82
JAG1	Jagged 1	0.66 ± 0.17	0.36

(*Continued*)

Gene Symbol	Gene Name	Fold Change (TGLI1 vs. GLI1)	*p*-value
KDR	VEGFR2	1.10 ± 0.45	0.79
LAMA5	Laminin alpha 5	2.43 ± 0.67	0.13
MDK	Midkine (neurite growth-promoting factor 2)	0.65 ± 0.23	0.33
MMP2	Matrix metallopeptidase 2	2.65 ± 1.03	0.14
MMP9	Matrix metallopeptidase 9	5.77 ± 3.16	0.59
NOTCH4	Notch 4	1.02 ± 0.27	0.92
NRP1	Neuropilin 1	0.89 ± 0.24	0.55
NRP2	Neuropilin 2	0.94 ± 0.34	0.68
PDGFA	Platelet-derived growth factor alpha polypeptide	1.36 ± 0.11	0.12
PECAM1	Platelet/endothelial cell adhesion molecule	2.93 ± 0.92	0.13
PLAU	Plasminogen activator, urokinase	1.78 ± 0.33	0.08
PTGS1	Prostaglandin-endoperoxide synthase 1	0.71 ± 0.14	0.20
SERPINF1	Serpin peptidase inhibitor, clade F , member 1	0.93 ± 0.15	0.79
SPHK1	Sphingosine kinase 1	1.26 ± 0.47	0.93
STAB1	Stabilin 1	5.97 ± 2.68	0.07
TEK	TEK tyrosine kinase, endothelial	1.87 ± 0.31	0.11
TGFA	Transforming growth factor, alpha	0.62 ± 0.09	0.03
TGFB1	Transforming growth factor, beta 1	1.54 ± 0.74	0.77
TGFB2	Transforming growth factor, beta 2	1.18 ± 0.31	0.81
TGFBR1	Transforming growth factor, beta receptor 1	0.68 ± 0.07	0.51
THBS1	Thrombospondin 1	0.79 ± 0.20	0.39
THBS2	Thrombospondin 2	1.99 ± 0.63	0.27
TIMP1	TIMP metallopeptidase inhibitor 1	2.99 ± 1.11	0.06
TIMP2	TIMP metallopeptidase inhibitor 2	1.15 ± 0.52	0.90
TNF	Tumor necrosis factor	0.43 ± 0.20	0.18
TNFAIP	Tumor necrosis factor–induced proteins	2.30 ± 0.38	0.10
